# Mental well-being of children and adolescents during COVID-19: evidence from the Italian context and possible future developments

**DOI:** 10.3389/fsoc.2024.1387030

**Published:** 2024-07-10

**Authors:** Francesco Orazi, Federico Sofritti, Davide Lucantoni

**Affiliations:** ^1^Department of Economics and Social Sciences,Polytechnic University of Marche, Ancona, Italy; ^2^Department of Economics and Law, University of Macerata, Macerata, Italy; ^3^Centre for Socio-Economic Research on Ageing, IRCCS INRCA—National Institute of Health and Science on Ageing, Ancona, Italy

**Keywords:** mental wellbeing, COVID-19, younger people, social distancing, intersubjectivity, distance learning

## Abstract

The article aims to discuss the increased emergence of mental health problems among children and adolescents, as an outcome of the COVID-19 pandemic.The results of a research study conducted among various professionals, such as psychiatrists and psychologists specialized in childhood and adolescence, are presented. The study, which uses both qualitative and quantitative methods, investigates the main consequences of the physical social distancing measures undertaken by the Italian government during the pandemic. The results are in line with the main evidence highlighted by international research and underline the particularly negative effects of the pandemic emergency on the mental health of minors. It reports how the limitation of intersubjective relationships and the forced digitalization of relationships has triggered or caused the emergence of multiple and varied disorders of the psyche, also linked to the area of reference (e.g., metropolitan, urban o remote areas), the socio-economic and cultural fragility of families, as well as the presence of previous mental issues within them. Finally, the research emphasizes how the understanding and management of the psychic health of these population groups, also from a health organization point of view, will be crucial to address the medium and long-term effects of such emerging issues among younger cohorts.

## 1 Introduction

One of the negative consequences of calamitous events concerns their impact on the psyche and individual cognitive processes (Sorokin, 2010). The social isolation imposed by COVID-19, for example, showed how the pandemic engendered or slatented^1^ disorders of the psyche, affecting the mental health of the population, with political-institutional, economic, and sociocultural repercussions (Sofritti, 2022). In this regard, some studies have hypothesised the existence of coronaphobia: a specific complex of fears determined by the coronavirus prompted by infodemic (Arora et al., 2020).

Among the areas on which the pandemic has most impacted we find the mental and psychological health of childhood and adolescence. Indeed, these age groups have been particularly challenged by the situation of confinement: in general, 75% of mental disorders emerge in the adolescent phase (Kessler et al., 2007), very often precisely because of the higher levels of isolation and loneliness (Achdut and Refaeli, 2020).

Both teaching and face-to-face peer group relationships were abruptly transferred to digital media (Orazi and Sofritti, 2020). This caused a transformation of intersubjective communication^2^, which deprived these age groups of a fundamental part of their social life, decisive for the development of their personality and social identity.

From an international perspective, scientific research has confirmed how the pandemic emergency, in its most severe phase, has challenged the mental health of children and adolescents. Many research studies were conducted between 2021 and 2022 addressing the impact of the pandemic on young people’s mental health. One of the most investigated aspects concerns the consequences of closing educational institutions and adopting distance learning. This trend, combined with increased time spent online and on social platforms, resulted in many young people experiencing a heightened condition of loneliness and isolation, triggering increased anxiety and depression (European Education and Culture Executive Agency, 2022). In this regard, a study coordinated by the “Bambino Gesù” Children’s Hospital together with La Sapienza and Tor Vergata Universities showed that out of a sample of 1,084 subjects, the increase in time spent in front of a screen, compared to the pre-pandemic period, affected 68.7% of children and young people overall. Exposure time appears to have more than tripled for school purposes (from just under an hour a day to three and a half hours) affecting 72% of children and young people, while recreational use almost doubled (from one and three-quarter hours to three hours) and affected 49.7% of subjects.

In addition, COVID-19 has had a significant impact on adolescent distress (Save the Children, 2022). The World Health Organization (2022) points out that in the 10–19 age group, one in seven teens suffers from a mental disorder and that suicide is the fourth leading cause of death in 15–19-year-olds. A study published in March 2022 (Armocida et al., 2022) analyzed trends in the causes of noncommunicable diseases that impact adolescents’ quality of life, pointing to mental disorders as the leading cause for the onset of a disabling condition (YLD, Years Lived with Disability) and noting that from 1990 to 2019, Years of Life Lost (YLL) due to mental illness had increased by 30%. World Health Organization (2022) also estimated a 25% global increase in anxiety and depression in 2020, pointing out that resources devoted to mental health were scarce: governments around the world spent an average of just over 2% of their health budgets in 2020, and many low-income countries reported having fewer than one mental health worker per 100,000 people. In this sense, the pandemic revealed a historic underinvestment in mental health services (World Health Organization, 2022).

Findings from both quantitative and qualitative studies confirm the predominantly negative consequences of the emergency in terms of isolation and interpersonal tension, as well as generally worsened mental health (Bell et al., 2023). The results of various studies on young people converge in identifying a largely negative impact of the pandemic on work, study, extra-professional life, and mental health (Hawke et al., 2020; Newlove-Delgado et al., 2021).

The Italian picture appears to be in line with international research evidence as the issue of the impact of the emergency on mental health has been raised by the Italian National Institute of Health since May 2020 (Istituto Superiore di Sanità, 2020). Some data attest to the impact of the emergency situation in this regard: the mental health index compiled by the Italian National Institute of Statistics (ISTAT) shows that the pandemic has strongly affected the mental health of the 14–19 age group, which dropped by almost 4% age points between 2020 and 2021 (OpenPolis, 2022). In contrast, this index shows a trend of stability for all other age groups over the same period (ISTAT, 2022). ISTAT also shows a different impact concerning gender: the drop in this index was more pronounced among adolescent girls (5% drop), than among adolescent boys (−2.4%); the same applies to the personal satisfaction indicator.

This issue was later confirmed by a research study, still in progress, conducted by the Child and Adolescent Authority in collaboration with the Italian National Institute of Health (AGIA-ISS, 2022). The initial part of the research, qualitative in nature, involved 90 afferents from both the health and social-health areas: child neuropsychiatrists, social workers, psychologists, pediatricians, teachers and school administrators. Among the major issues that emerged were: sleep and eating disorders, thoughts of suicide, attempted suicide, self-injurious behavior, and social isolation. Prolonged use of technological devices can be considered as a factor associated with the risk of occurrence of some of these disorders. For example, the phenomenon of hikikomori^3^ (among cases of social isolation) is often associated with internet addiction (Pozza et al., 2019), just as time spent in front of a screen in the evening hours may play a role in the development of sleep disorders (Moavero et al., 2023). In addition to this, as far as education is concerned, an increase in learning, attention and language disorders has been detected, which can also and especially be attributed to distance learning (Champeaux et al., 2022). In this regard, previous studies indicate that screen exposure leads to structural changes in the brain (Loh and Kanai, 2016; Jha and Arora, 2020), such as reduced volume of the cortex with loss of integrity of the white matter region (Takeuchi et al., 2018). These alterations entail reduced attentional competence, processing speed and verbal intelligence. In addition, the most common online tasks (such as searching and reading) reduce the functional connectivity of regions around the temporal gyrus, responsible for long-term memory formation and retrieval of learned material (Liu et al., 2018). Also, cognitive and emotional conduct disorders, uncertainty about the future and a state of generic frustration, together with an increase in the use of psychoactive substances and alcohol have been found (AGIA-ISS, 2022). As a result, professionals have experienced a marked increase in requests for help and intervention, which has lengthened waiting lists in public facilities. For this reason, many families have been forced to turn to the private sector, with a major impact on family budgets and socioeconomic inequalities.

Data on hospitalizations confirm this national trend: in 2022 the Italian Society of Child and Adolescent Neuropsychiatry (SINPIA) showed that there were 394 beds in child neuropsychiatry wards across the country (even absent in four regions: Calabria, Molise, Umbria and Aosta Valley); a limited number and totally insufficient to cope with what, following COVID-19, has become a real emergency. Almost a quarter of the total number of children and adolescents show signs of anxiety and depression disorders (Panorama della Sanità, 2022).

Hospital admissions for symptoms attributable to psychiatric disorders were already on the rise in the decade prior to the pandemic and, between 2021 and 2022, exceeded pre-pandemic admissions (Bortoletto et al., 2022; Panorama della Sanità, 2022; Marin et al., 2023). By September 2021, the number of children and adolescents hospitalized for psychiatric disorders had already surpassed that of 2019. As noted by Save The Children (2022) (Pulcinelli and Pistono, 2022), hospitalizations for neuropsychiatric disorders grew by 39% between 2019 and 2021. The top two causes of emergency department admissions for neuropsychiatric pathology were psychosis and eating disorders. For admissions to the ward, the leading cause was suicidal ideation.

More than three-quarters of ward admissions occur in emergencies, and patients show clinical pictures of increasing complexity. In some regions, children are admitted to adult psychiatric wards due to the absence or insufficiency of beds in neuropsychiatric wards. Territorial facilities also point to structural deficiencies: in 2020, a quarter of patients said they had difficulty accessing these facilities, and technological deficiencies caused problems concerning ensuring continuity of care during confinement. This reflects not only structural deficiencies in technological equipment (Sofritti and Orazi, 2020), but also critical organizational issues due to poor implementation of territorial services that the National Health Service historically suffers from (Sofritti, 2021). This, in fact, takes place in a context in which the resources available to public Mental Health Services are continuously decreasing, standing at under 3% of the national health fund, while the European indication is 10% for higher income countries. This is a very distant target even from the minimum standard of 5%, taking into account that in Italy from 2018 to 2020 this share was reduced from 3.8 to 2.75%. This led to a decrease in the number of Mental Health Departments (MHDs) from 183 in 2015 to 141 in 2020, which was accompanied by a significant decrease in medical staff: it is estimated that by 2025 there will be a further decrease of about 1,000 psychiatrists (Istituto Superiore di Sanità, 2020).

Based on these assumptions, the paper presents the results of a research study regarding the effects that the pandemic emergency has caused on the mental and psychological health of children and adolescents in Italy. The research, conducted through in-depth interviews and a web survey, was addressed to a sample of child neuropsychiatrists and developmental psychologists. The objective of the study is twofold: to investigate the views of experts (key-informants) in the field of mental health; and to produce an overview of the effects of the emergency on the mental health of children and adolescents. The aim is to reconstruct the picture of the Italian situation, also in the light of the international context, in order to draw indications for future mental health policies, highlighting the economic impacts of its deterioration at the territorial and social level.

The paper presents the following articulation. Section 2 describes the research methodologies and materials used. Paragraph 3 presents the most relevant results of the research focusing on: the effects produced by social distancing on children’s health; the impact of distance learning on intersubjective dynamics in school contexts; the socioeconomic effects of the pandemic on territorial realities; and finally sketching future scenarios that the pandemic could determine in the medium term on children’s psychological health. Finally, an attempt will be made to highlight the main evidence arising from the research.

## 2 Materials and methods

The research was carried out between March and September 2022 and consisted of two levels: a first qualitative phase involving semi-structured interviews with privileged witnesses, i.e., professionals working in the field of neuro-psychiatry and developmental psychology; the second quantitative phase involved a web survey among psychiatric physicians and psychologists working in hospital and community settings.

The first phase was exploratory, aimed at discussing with professional figures the most relevant issues raised by the pandemic emergency and lockdown concerning the mental health of children and adolescents. In this phase, the most relevant points and crucial issues to be investigated through the web survey were identified, determining the analytical dimensions to structure the questionnaire.

The panel of neuropsychiatrists and psychologists used in the exploratory phase was located at the ‘Salesi’ pediatric hospital in Ancona. The second phase was preceded by mapping the child neuropsychiatry departments of the main regional hospitals in Italy, reconstructing a national picture. At the same time, email contacts of psychiatrists and psychologists were collected from the websites of the identified health facilities, to which the questionnaire was sent to be filled out online. The web survey is an established technique in social research and has been adopted for many years now (Snelson, 2016; Stern et al., 2017). In order to achieve a snowball effect (Leighton et al., 2021), in the invitation e-mail it was also asked to disseminate the questionnaire as widely as possible to colleagues and facilities that could be useful for research purposes.

The sampling design is of the reasoned type. The sample consisted of 190 practitioners selected from 30 national paediatric hospitals. The response rate covered 35.8% of the sample (68 professionals). The methodological choice was coherent and appropriate due to the low numerosity of the statistical population (*N* = 190), which made it possible to use the theoretical saturation procedure. Concerning the reliability of the sample, we accepted a margin of error of ±10%, with a confidence interval of 95%. The object of analysis and the area investigated meant that the expertise and knowledge of the research participants qualified the study more than mere statistical representativeness.

The structure of the questionnaire reflects the main analytical dimensions identified from the analysis of national and international literature on the topic (Achdut and Refaeli, 2020; Newlove-Delgado et al., 2021; European Education and Culture Executive Agency, 2022; Moavero et al., 2023), as well as what emerged from the semi-structured interviews in the exploratory phase. The first part concerns the general information of the participants. In particular, in addition to basic sociographic data and the regional reference areas, organizational aspects were also considered of interest: the role covered within the afferent structure (Director of Complex Structure, medical manager, psychologist manager, contracted freelancer, resident etc.) and the organizational scope of their structure (hospital, territorial facilities, private practice). In this section, an item related to the type of territorial context in which each professional operates was also introduced: large, medium, small town or rural area. It is believed, in fact, that this variable may have played a major role in the presence of a “total social fact” such as a pandemic (Orazi, 2021; Orazi and Lucantoni, 2021) and under special conditions such as those of large-scale confinement.

The second part of the questionnaire explores the possible link between social distancing and the onset of mental disorders. The third focuses on family issues and aims to investigate the relationship between structural family conditions (socioeconomic and family background, regardless of the pandemic emergency) and their possible role in triggering disorders. An additional dimension of analysis in the questionnaire is the educational aspect and distance learning, especially about its possible impact on learning disorders. Another section of the questionnaire deals with the type of disorders that participants thought the emergency situation could have contributed to or exacerbated. Finally, the perspective of professionals concerning the impact of the pandemic on the mental health of children and adolescents in the future was investigated.

A total of 68 questionnaires were completed; the main findings will be presented in the next section.

## 3 Results

In this section the main results of the survey are presented in three different parts: first, a demographic and professional overview of the sample is provided. After that, two main aspects are addressed: the impact of social distancing and the socio-economic impacts of the psychological changes prompted by the pandemic.

### 3.1 Demographic and professional profile

The sample is rather heterogeneous in terms of age: in fact, there are various age groups present, the most represented being 45 to 55 years old (Table 1).

**Table 1:** Demographic and professional profile of the sample*.

Age groups:	25–34	35–44	45–55	56–65			
	10,34%	37,94%	44,82%	6,9%			
Organizational role:	Medical managers	Freelancers	Postgraduate students	Complex facility directors	Simple facility directors	Psychologist managers	Contracted specialist physician
	51.7%	10.4%	10.4%	10.4%	6.8%	6.8%	3.5%
Working facility:	Hospital	Territorial facilities	Private outpatient				
	51.7%	37.9%	10.3%				

Area(s) of reference:	Large-sized urban areas (over 300,000 inhabitants)	Medium-sized urban areas (between 100,000 and 300,000 inhabitants)	Small-sized urban areas (less than 100.000 inhabitants)	Areas with less than 50,000 inhabitants
	44.8%	57.1%	10.7%	3.6%

*This table shows the distribution of the sample by age groups, organizational role held, the type of facility and territory in which they work. Source: Our own elaborations on data collected through the “Questionnaire on the Effects of Social Distancing for the Mental Health of Children and Adolescents”.

This is indicative of a target group of participants who have already gained a fair amount of professional experience in the field, and thus a high level of expertise. From an organizational role perspective, just over half of the participants (about 52%) are medical managers in the psychiatric field, while about 10% are postgraduate students. A similar proportion concerns the complex facility directors, who hold management roles (directors of hospital departments). Other figures, however, are residual. In general, therefore, the majority of participants are neuropsychiatrists, while the proportion of psychologists is smaller.

Territorially, Emilia-Romagna is the region from which the largest number of responses was received, followed by Marche and Liguria. The most represented province was Genoa. The majority of respondents come from hospital facilities (about 52%), nearly 38% from territorial facilities, and the remainder from private practices. About 45% of the respondents work in large-sized urban areas (over 300,000 inhabitants), about 38% in medium-sized urban areas (between 100,000 and 300,000 inhabitants), while a smaller number of participants work in entities with less than 100,000 inhabitants. Most of the professionals who participated in the survey practice in hospital facilities (51.7%), 37.9% operate within public territorial facilities, while 10.3% operate exclusively in private practices. Medical executives work 46.6% in hospital facilities and 53.4% in territorial facilities; it should also be noted that among freelancers about one-third also work in hospital facilities, while complex facility managers are more concentrated in territorial facilities (66.6%) rather than in hospitals (33.4%).

### 3.2 Social distancing

The issue of social distancing, as already highlighted by analyses regarding its negative impact on the adolescent population, especially in terms of mental and behavioral health, raises several questions.

The sample of neuropsychiatrists analyzed confirms that social distancing has caused a lengthening of waiting lists (75.9%) and concomitantly a general increase in cases and emergency interventions (86.2%), which in 89.3% of cases affected the adolescent population. Moreover, as other studies and analyses highlight, our survey also confirms a causal impact relationship between social distancing and onset of disorders and/or pathologies among minors, as expressed by 86, 2% of the surveyed neuropsychiatrists. Among them, 73.9% consider that the greatest impact of social distancing is the alteration of adolescents’ normal daily relationships. This mechanism would displace them in their relational and socio-affective habits.

In some paradoxical ways, the very age cohorts most accustomed to using social networks and educated in long-distance relationships have suffered most from the negative effects of distancing.

To the question which included the possibility of giving three answers about how social distancing had produced a change in pathological outbreaks among minors before the pandemic, the sample shows an evaluative difference between child and adolescent populations. In the former, the occurrence of new pathologies/disorders due to lockdown is answered positively by 57.1% of the respondents, while for the adolescents this figure “spikes” to 88.2%. The pathologic onset among the child population mainly concerned sleep disorders (50%) and children’s demotivation and fatigue (43%), while among the adolescents the sample highlighted more serious issues: self-harm gestures (predominantly cuts) (76%), suicide attempts (72%), difficulties in school reintegration (48%), relational disorders (32%).

However, the extent to which these described dynamics mix the digital transposition of socio-affective relationality and the socio-economic conditions of families, where cases of child and adolescent distress most frequently occurred, is not lost on us. Indeed, our analysis shows that the emergence of disorders mainly concerns families with previous psychiatric outbreaks among their members, families characterized by forms of social marginality, and families affected by economic difficulties both before, during and after COVID-19. Less significant, however, is the incidence in families with separated/divorced parents and in those with low parental education and low availability of cultural resources both cognitively, materially and technologically. Forced and prolonged cohabitation in enclosed household spaces was also not a crucial/critical/important issue. In contrast, 78.6% of the sample saw that it was often the parents who were the main vectors of anxiety towards their children, just as 69% saw a significant increase in attachment problems/disorders between parents and children (for the assessment of which, it was possible to provide three responses for each modality in the questionnaire), the main evidence of which included self-harming gestures (88%), suicide attempts (68%), severe eating disorders (56%).

As can be seen from the data, practitioners (territorial child neuropsychiatrists) show a greater tendency to impute to parents the transmission of anxieties and fears to their children (81.8%) versus professionals working in hospitals, which attest to this result at 71.4%. It should be noted that the private outpatient component, while representing a minority share of the sample, ascribes 100% of this responsibility to parents. Taken together, these results reveal a problematic picture of family intersubjectivity, that is, how generations confront each other about the meaningful structures that govern their actions and behavioral orientations and the often-uncommunicative languages that the pandemic and social distancing have exacerbated.

### 3.3 Socioeconomic impacts of COVID-19

The social, cultural and psychological transformations triggered by the pandemic have impacted various social and economic dimensions. Distance learning, for example, was the main emergency measure adopted to ensure the continuity of education in universities, primary and secondary schools. This sudden reorganization of teaching has raised several critical issues, including the need to adapt the relationship between teachers and students, as well as the traditional pedagogical approach, within new technologically mediated learning contexts. Some implications of these critical issues were evaluated by the sample of experts who participated in the survey in order to investigate the possible relationship between distance learning and the occurrence of diseases and disorders in adolescents and children during the pandemic.

On a rating scale of 1 to 5, the incidencee of new pathologies/disorders in children as a result of distance learning has an average of 3.1. The respondents believe that demotivation and fatigue are the most recurrent disorders (4), along with relationship disorders (3.9). This also seems to have negatively affected the school reintegration process, and the recurrence of dropout cases (3.7), while the recurrence of learning and somatoform disorders (characterized by physical symptoms of a chronic nature, worry and difficulty in performing everyday activities related to these symptoms) (3) is less significant, although not negligible. However, among the disorders and pathologies examined, the least recurrent ones seem to be memory disorders (Table 2).

**Table 2:** Recurrent disorders/pathologies in minors as a result of distance learning*.

Main diseases/disorders	Average (μ)
Demotivation and fatigue	4
Relationality disorders	3.9
School reintegration/abandonment	3.7
Sleep disorders	3.3
Learning disorders	3
Somatoform disorders	3
Memory disorders	2.7

*Sample mean judgment on recurrence of major disorders/pathologies in children as a result of distance learning. Source: Our own elaborations on data collected through the “Questionnaire on the Effects of Social Distancing for the Mental Health of Children and Adolescents”.

On the level of socialization, distance learning thus seems to have produced negative effects by hindering the development of customary mechanisms capable of regulating social relationships, promoting the onset of forms of anxiety and refusal to return to school.

A further consideration of the topic comes from the analysis of the results presented in the previous paragraphs regarding the strong impact of the socioeconomic and cultural fragility of families on the development of pathologies and disorders by adolescents and children during the pandemic (Sec. 5.1). In particular, the forced experimentation of distance learning seems to have increased the gap between students with greater cognitive and economic resources and students who are less endowed with such resources, amplifying the already marked inequalities in traditional schooling. In this sense, using technological devices to convey education entails not only investment on the part of the school system but also the concomitant removal of previous sociocultural problems that run through the relationship between educational institutions and families (Orazi and Lucantoni, 2021).

Moving on to analyzing the territorial context, 57.1% of respondents consider it to be a relevant factor in the occurrence of post-Covid psychological problems among the population of minors. Within this share, the metropolitan area is the most relevant with 69.3% of practitioners evaluating it as a problematic source on the psyche of minors. The percentage decreases to 46.7% for urban areas and 33.4% for rural ones. Very interesting, finally, is the operators’ evaluation (86.2%) which shows the belief that in recent years neuropsychiatric activity has increasingly shifted to the field of psychiatric problems, underestimating the psychotherapeutic and relational aspects with patients. According to the sample (96%), this shift has been poorly governed in terms of organization and management with investments which are deemed inadequate. Besides the lack of dedicated beds and regionally widespread facilities (Sec. 3.3), the main problem is that after hospitalization families rarely benefit from adequate support. In the local health services [namely, Local Health Board (ASL)], there is no child neuropsychiatry care network that does prevention and early referral for treatment. It is underestimated that most mental disorders begin in the developmental age and that the training of physicians should be reorganized according to this evidence/emergency. These are organizational, technical and structural delays that need to be addressed in the short term by maturing an awareness of how adolescent age is a source of new and complex pathological issues.

In the medium and long term, the scenarios drawn (in terms of the plausibility, on a scale of 1—very likely—to 3—unlikely of the proposed items) by the sample on the effects of social distancing, distance education, and government strategies to deal with the spread of neuropsychiatric disorders and pathologies among adolescents and children highlight multiple aspects. Respondents believe it is very likely (60%) that the disorders that have emerged with social distancing will show their most acute effects in the next 5 years, and that these will have significant effects on the quality of children’s education (67.9%—very likely). However, concerning the quality of education, the sample’s opinion regarding the possible lasting effects of the pathologies/disorders that have arisen with distance learning appears to be less unanimous, as 46.4% believe that the occurrence of such effects is likely, while 28.6% believe it to be unlikely. Ultimately, the onset of psychological disorders in adolescence seems destined to occur increasingly early (very likely according to 85.7% of respondents). This aspect is part of an Italian context where the resources available to public Mental Health Services are steadily declining, standing at under 3% of the national health fund, while the European indication is 10% for higher income countries.

The results of our study point to the need for organizational restructuring of territorial deanships and departments of child psychiatry (92.9% – very likely), aimed at absorbing the expected increase in the demand for care services, including expanding the number of available beds (82.1%—very likely) and training new profiles of the child neuropsychiatrist to cope with the growing criticality induced by COVID-19 (67.9% – very likely). These critical issues need to be addressed from a systems perspective, not only from a health point of view but also in terms of integration between social, health and school services to monitor, prevent and intervene in cases of growing distress (85.7%—very likely). However, from the scenario analysis, it is only 39.3% likely that over the next five years political institutions will invest increasing resources in this area.

## 4 Discussion

The findings of this study appear to be in line with national and international debate and highlight how social distancing has eroded normal human intersubjectivity and how this limitation has reverberated negatively on the population of minors (Hawke et al., 2020; Newlove-Delgado et al., 2021; Bell et al., 2023). In particular, this has affected one of the major changes the new generation undergoes, namely the lack of “intergenerational connection” whereby young people have fewer comparisons and support from competent adult role models (Fonagy et al., 2022). National and international research has shown the extent to which social distancing and distance learning have together represented interesting and displacing novelties for large portions of the population. It is within the framework of this displacement that one can understand the marked increase in psychological distress among minors, especially regarding adolescence. Consistently with the framework outlined by national and international research, our study places at the center the question of intersubjectivity and what it means to interrupt or alter its normal routine, as happened during the lockdown. Indeed, the pandemic has imposed a radical shift in the modes of intersubjective transmission of meaning, transferring online a very substantial part of the external relationships normally experienced through the joint presence of interlocutors. This has imposed a technological mediation that has shown the increasing prominence of non-presence relations, especially in interaction dynamics previously experienced through face-to-face relations (e.g., didactics at a distance) (Williamson et al., 2020). The research results highlighted multiple aspects.

First, restrictions to cope with the pandemic seem to have had a particularly negative impact on adolescents, for whom there has been a significant increase in the development of neuropsychiatric disorders and pathologies (self-injurious gestures, suicide attempts, and difficulties with school reintegration). This phenomenon seems to be amplified by the fragility of family ties/bonds—deterioration of socioeconomic and cultural conditions, presence of previous pathologies in the family history—and by the area of origin, particularly for those living in metropolitan areas. The area, as a relevant factor in the development of neuropsychiatric disorders and pathologies among adolescents (Chen et al., 2020), is also overlaid by the Internet, which has fueled individuals’ need for relationality on the one hand and the attempt to ensure the continuity of public and private services through a sudden process of digitization on the other (Lin et al., 2023). In this sense, distance learning has been the most relevant experiment in digitizing child relationships, showing how the amputation of the social and relational dimension also negatively affects learning processes.

Second, these repetitive relational practices of non-presence also involve the consolidation of a new configuration of the body within the social world (Kelly, 2017). The body seems to be reduced to a passive function of supporting the technological tool and generating new forms of discomfort. The removal of the body from the intersubjective dimension of learning also affects students’ ability to adapt to its progressive nature, as the same requires processes of sedimentation of skills that find implementation not only in mnemonic exercise but also through the invention of gestures that are inextricably linked to the sign they produce, for example in the case of freehand writing. Such gestures determine the creation of a personal learning style that technologically oriented teaching should be able to promote in turn (Orazi and Lucantoni, 2021).

The two aforementioned points have mutual implications concerning the way the body and relationships have been affected by the pandemic and can be argued in connection with the intensive use of digital devices that followed.

In particular, to properly frame the data, it is worth emphasizing that the lockdown has forced a huge mass of routine relationships normally carried out offline to be transferred to the internet: from distance education to ways of experiencing leisure time and communication with peer groups. That it was the adolescents who suffered most from this “sudden change of world” is, in our opinion, only an apparent contradiction: systematic training in digital technologies and long-distance relationships is not sufficient to consider these processes as neutral, especially when they, as in the case of the pandemic, have become totalizing. From this perspective, the first question concerns the ways in which young adolescents interweave face-to-face and technologically mediated intersubjectivity. Such intermingling, even if it can be creative and enhance relational density, shows, or rather showed during the lockdown, the limitations associated with the hypo-sociality of technologically mediated relationships (Paccagnella, 2001). Despite advances in digital technology, online intersubjective relationships still exhibit a marked hypo-sociality that is only minimally mitigated by the possibility of symbolic integration made available by web languages (e.g., emoji).

In physical relationships, interlocutors experience body language, gestures, postures, changes in vocal tone, and glances, all those aspects that constitute the so-called meta-communication. The same is crucial in making intersubjectivity dense and significant. If in communicating a concept I adopt a high or low tone of voice, a look of openness or closure/censorship towards the other, this integration of bodily communication concerning the expressed concept changes its meaning and receptive quality to the interlocutor. In online relationships, on the contrary, this meta-communication is re-metaphorized by the technical medium.

The impossibility of physical interaction and the rarefaction of meta-communication have made many people experience the distance of external contact in spaces cramped by imposed domesticity. This circumstance opens up the second question of our work: the aspects of fragility that a technologically oriented intersubjectivity can bring about, especially in adolescent-aged individuals.

During adolescence, for example, the rapid development of social-affective circuits increases the need for social rewards and feelings of concern about peer group judgment. Important adolescent goals are to establish meaningful relationships with that group, gain independence from adults, and explore one’s identity (Cicchetti and Rogosch, 2002). Social media refocuses these adolescent tasks: the peer group is constantly available, personal information is in the public domain, and judgment is instantaneous and quantifiable through “likes” and “views” (Nesi, 2020).

The experience of lockdown, with the enormous difficulty encountered especially among adolescents in combining their digital forms of life with the traditional forms of intersubjective contact, shows the profound limits of a type of communication seemingly open to the world but in fact confined to the domestic space of a video, an aspect that more than others has contributed in our opinion to the disruption of mental health in many adolescents.

Moreover, the emergence of new disorders and pathologies and the slatentization of pre-existing ones, as possible consequences of distance learning, should also be interpreted in light of the broader pathway of channeling and rationalizing the development not only of skills, but also of the individual’s affective and relational development through the use of digital devices. Indeed, studies on the neuroplasticity of the brain highlight that digital tools are not just prostheses; on the contrary, they stimulate or inhibit specific mental activities, shaping the structure and functioning of the mind (Johnson, 2006). According to this perspective, the split of attention required by multimedia products may reduce the capacity for learning and comprehension (Rockwell and Singleton, 2007). Concerning the affective and relational sphere, Nass and Yen (2012) argue that good emotional development requires relational practices where that part of the brain that presides over emotionality is enhanced, that is, the reevaluation of face-to-face relationships, which are being increasingly surrogated by those at a distance. The brain must train itself to pick up on even imperceptible changes in another’s face and vocal intonation. Empathy and sociality require continuous and spontaneous practice and reciprocal interpretations in the vital worlds in which socialization is built. Communication and digital interactions are from this point of view a kind of “castration”.

This has profound policy implications, as the results also highlight the need for an increase in the number of beds in child neuropsychiatry wards, as well as their creation in the four Italian regions which lack them. On this front, there are marked gaps at the European level. According to Eurostat (2020), there were an average of 73 psychiatric care beds per 100,000 inhabitants (14%) in Europe in 2018, down from 79 in 2004. National data reveal a diverse and uneven domestic picture. Belgium has 135 places, Germany 128 and Latvia 122. At the bottom of that ranking are Ireland (34 places) Cyprus (18) and Italy with only 9 psychiatric care beds per 100,000 inhabitants. In the Italian context, at the territorial level, there is a need to strengthen the prevention network by increasing the number of pediatricians: Save the Children estimates that at least 1,400 are needed to ensure a widespread service; similarly, there is a shortage of daycare centers and residential services for adolescents with psychiatric disorders (Pulcinelli and Pistono, 2022). In addition, only 12% of public health spending is allocated to prevention and primary care medicine: an increase in this, also in reference to what is envisaged in the PNRR, would be necessary to meet the growing mental health needs of childhood in the medium and long term.

Finally, there is a need for targeted interventions aimed at increasing the psychological capital of individuals (Alat et al., 2023), especially in the training and educational spheres, enabling the management of young people’s mental health through awareness-raising actions at crucial stages of growth. Measures such as the psychologist bonus, introduced in Italy with Law 15/2022, are indicative of an awareness of these issues and allow to overcome the stigma attached to mental health problems. Of the more than 300,000 Italians who have applied for the grant, 43.55% of applicants are in the 18–35 age group, while 16.62% are minors up to the age of 18 (European Education and Culture Executive Agency, 2022).

In this sense, educational and informational interventions aimed at making young people more aware/conscious of the delicacy of the phase they experience, especially in adolescence, could foster more appropriate management of mental health by institutions (Slade, 2010). This should be instrumental in making families more aware, particularly those at risk, by detecting latent symptoms early and avoiding overmedicalization^4^ of disorders of the psyche (Fisher, 2009).

This study suffers from a number of limitations: the first related to the sample of survey participants, who are all part of professional groups dealing with child and adolescent mental health. Their perspective, however knowledgeable and specialized in the field, should be integrated with that of the patients themselves and their families in order to obtain a multi-dimensional picture of the phenomenon and the policy interventions to be implemented. Secondly, the study was conducted in a national context: the involvement of multiple national contexts would enable the acquisition of a more comprehensive and complex perspective, providing elements for comparison on an international scale and an assessment of possible strategies to be adopted at the community level.

Finally, the research opens up further questions and areas for study in the relationship between pandemic (distancing practices) and mental health: in particular, it would be interesting to investigate the consequences of emergency in the medium and long term. This could allow analysis of the impact of distancing over a significant period of time. The medium- and long-term analysis would show the greater or lesser coping capacities from the perspective of both individuals and institutions, with particular reference to the ability to manage health issues and related policies.

## Data availability statement

The raw data supporting the conclusions of this article will be made available by the authors, without undue reservation.

## Ethics statement

The studies involving humans were approved by Research Ethics Committee of the Università Politecnica delle Marche. The studies were conducted in accordance with the local legislation and institutional requirements. The participants provided their written informed consent to participate in this study.

## Author contributions

FO: Conceptualization, Formal analysis, Investigation, Supervision, Writing – original draft, Writing – review & editing. FS: Formal analysis, Investigation, Methodology, Writing – original draft, Writing – review & editing. DL: Data curation, Formal analysis, Investigation, Writing – original draft, Writing – review & editing.

## Footnotes

^1^The term indicates the emergence of that which is latent, such as a past negative event, a trauma that has not been overcome and metabolized, capable of causing the triggering of symptoms such as anxiety, panic, and modes of thinking that bring discomfort, discomfort, and pain.

^2^Intersubjectivity refers to the communion of subjectivities that makes possible an objective world produced by the conventions adopted by a community. Intersubjective communication is the medium for the emergence of all knowledge and for all possible forms of agreement between generations (Muzzetto, 2014).

^3^The term ‘Hikikomori’ literally means ‘staying apart’ and is used to indicate those who decide to withdraw from social life for long periods (from a few months up to several years), closing themselves at home, without having any direct contact with the external world, sometimes not even with their own parents.

^4^Medicalization consists of defining a problem in medical terms, using medical language to describe a problem, adopting a medical framework to understand it, or using a medical intervention to “treat” it (Conrad, 1992). This, without taking into account the cultural, relational and ideological factors that outline the traits of the society in which mental pathologies emerge (Fisher, 2009).
